# Spacious Environments Make Us Tolerant—The Role of Emotion and Metaphor

**DOI:** 10.3390/ijerph181910530

**Published:** 2021-10-07

**Authors:** Chenjing Wu, Fuqun Liang, Xiaoling Liang, Chuangbing Huang, Hua Wang, Xianyou He, Wei Zhang, Don Rojas, Yan Duan

**Affiliations:** 1 Key Laboratory of Brain, Cognition and Education Sciences, Ministry of Education, China, School of Psychology, Center for Studies of Psychological Application, and Guangdong Key Laboratory of Mental Health and Cognitive Science, South China Normal University, Guangzhou 510631, China; wuchenjing@m.scnu.edu.cn (C.W.); 2019023118@m.scnu.edu.cn (F.L.); 2019022982@m.scnu.edu.cn (X.L.); 2020023405@m.scnu.edu.cn (C.H.); 2019022987@m.scnu.edu.cn (H.W.); psycheungwai@163.com (W.Z.); 2019022973@m.scnu.edu.cn (Y.D.); 2 Department of Psychology, Colorado State University, Campus Delivery 1876, Fort Collins, CO 80523, USA; don.rojas@colostate.edu

**Keywords:** spacious environment, narrow environment, moral judgment, emotion, metaphor

## Abstract

The physical environment plays an important role in moral cognition. Previous research has demonstrated that the physical environment affects individual moral judgment. Investigators have argued that the environment influences moral judgment through emotion and cognition, such as during metaphor processing. Following the intensification of urbanization and increases in population size, the phenomenon of a narrow environment has become more common. However, the relation between environmental spaciousness and moral judgment has not been thoroughly examined. We examined the effect of environmental spaciousness (spaciousness vs. narrowness) on moral judgments in Experiment 1 and Experiment 2. Results showed that participants report a higher rating score of moral judgment in more spacious environments compared with narrow environments. We further explored the roles of emotion and metaphor in the relation between environmental spaciousness and moral judgments. We found support for a partial mediation effect of emotion in the relationship between environmental spaciousness and moral judgment. The results also supported an association between the concept of spaciousness and tolerant cognition. Spacious environments may elicit positive emotions and more tolerant cognition, which in turn influences moral judgment. These results provide new evidence for the influence of the environment on moral judgments, and more attention may be warranted to incorporate this relationship in environmental design.

## 1. Introduction

Environmental psychology shows that the environment we live in affects our health and cognition. The width and narrowness of the environment are very important attributes of the environment. However, there is a lack of corresponding research on the effects of environmental width and narrowness on individuals. Therefore, the current study focuses on the effects of perceived environmental spaciousness on individuals, as well as several mediating factors.

Studies have shown that the physical environment has an important effect on our cognition, behavior, and health [1,2]. Narrow spaces are defined as limited spaces that are relatively isolated from the outside world, with restricted access and poor natural ventilation. Narrowness and spaciousness are important attributes of the environment. Spacious environments as opposed to narrow ones, are spaces with larger horizontal distance and wide range of view. In recent years, with the increase in population and the intensification of urbanization, the per capita area has been getting smaller and smaller in many cities, suggesting a trend towards narrowing. Capsule housing has even emerged in some places and has been somewhat sought after. Research found spacious environments offer more affordances (opportunities for action), and conversely, narrower environments should offer fewer affordances from an ecological psychology perspective [3]. Though such small or cramped spaces are becoming more common, it is relatively unclear what impacts such environments have on cognition. 

In social cognition, moral cognition is an important component. Moral cognition and moral behavior are positively related, for example, when ratings of moral judgments are evaluated against moral actions [4]. Exploring the effect of environmental spaciousness on moral judgment was an important problem. Moral judgment has always been an important concern, being defined as an evaluation of a person’s behavior or characteristics based on a set of cultural or culturally defined virtues [5]. The physical environment plays an important role in moral judgment. Research suggests that with changes in physical environments are associated with changes moral judgments [6,7,8,9,10]. Schnabel et al. explored the role of the environment in moral judgment [11]. They found that after being exposed to a disgusting environment, participants’ unfavorable attitudes towards immoral issues increased. Similarly, an unclean environment can increase the severity of moral judgment compared with clean conditions [12]. Zhou et al. explored the effect of the environmental brightness and darkness, and found that people tended to judge the protagonist of immoral stories and moral dilemmas as more immoral under brighter light conditions [13]. As another attribute of the environment, we propose that environmental spaciousness will exert an influence on moral judgments. In the current paper, we first examined the effect of environmental spaciousness on moral judgment in Experiments 1 and 2.

Research also suggests that different physical environments can trigger different emotions [14] and that emotion plays an important role in moral judgment [15,16,17]. For example, a beautiful environment was associated with positive emotion [18]. Positive emotions can lead individuals to make more tolerant moral judgments [19,20]. Valdesolo and DeSteno (2006) found an effect in their research, which asked participants watching a comedy or documentary to judge the appropriateness of the observed behaviors. The results suggested that participants watching comedy made more tolerant moral judgments compared to the participants watching the documentary [20]. Conversely, more negative emotions and environments initiating negative emotions (e.g., dirty, bad smells, air pollution) trigger stricter judgements [21,22]. Environments initiating negative emotions can also lead to greater judgements of immoral behavior, even for morally neutral events [6,22,23,24]. Researchers have explored the mediating roles of emotion in moral judgment [22], and have found that affective states influence the relationship between control deprivation and moral judgment [6].

Metaphor means using concrete, tangible, simple initial source concepts (size, weight, and brightness) to express and understand abstract, intangible, complex target domain concepts [25], such as the relation between darkness (simple concepts) and immoral behavior (complex concepts) [13]. Metaphor is always associated with individual experience, [25,26,27,28,29] and it can actively influence an individual’s thoughts and behaviors in a deeply sub-conscious manner. The metaphor was important in the relation between environment and morality [18,30,31]. For example, researchers found that physical factors, such as color, size, brightness, and distance, were related to moral judgements [32,33,34,35,36,37,38]. The brightness of the environment influenced individual morality, which found participants in slightly dimly lit rooms have more cheating behavior than those in well-lit rooms [39]. Environmental cleanliness has also been shown to have some implicit relationship with morality, with studies finding that fresh smells increase virtuous behaviors, such as reciprocity and charitable behavior [40]. Individuals exposed to a foul-smelling, offensive odor condition judged moral violations more harshly than individuals in the control group condition [11].

Spatial metaphors are a type of metaphor defined by constructing and understanding non-spatial concepts using spatial constructs within the origin domain [41]. Because of the inability of people to disengage from the space they occupy, researchers have also argued that of all metaphors, spatial metaphors occupy an important place in the human cognitive and abstract conceptual system [25]. In previous explorations of spatial metaphors, researchers have identified metaphorical relationships between the up and down properties of space and morality. Research has also found that cognition of words with different positive and negative meanings is influenced by spatial stimuli [34]. Spaciousness is a common physical property of the environment. In dictionaries, spaciousness or width refers to big distance, big acreage, and broad area. Spaciousness usually also implies a large carrying capacity and tolerance of things. This spatial property may influence tolerant attitudes toward behavior, i.e., the formation of implicit associations between spacious environments and tolerance. In certain cultures, concepts of spaciousness are used to describe tolerant cognition. For example, Chinese people often associate words depicting tolerant cognition, including ‘tolerant’ (kuan1rong2) and ‘loose’ (kuan1song1) ‘being lenient with others’ (kuan1yi3dai4ren2) (these words stand for tolerance judgment and tolerant cognition, respectively) with the conception of spaciousness or width (kuan1). In English, it is also common to see the association of extensiveness and tolerance as well as the association of narrowness with intolerant perceptions. For example, people often use words such as wide-views, broad-views, and broad-mindedness to describe tolerant cognitive attitudes, or the inclination to respect views and beliefs that differ from your own. People also use these words such as narrow-views and narrow-mindedness to describe an intolerant cognitive attitude, which indicates lacking tolerance or flexibility for different viewpoints.

**Figure 1:**
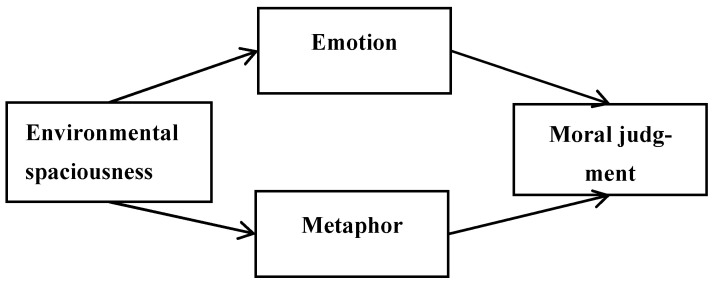
Hypothesized model depicting the relation between environmental spaciousness and moral judgment.

Combining the above literature, we suggest there are two possible paths by which environmental spaciousness can affect moral judgment: (1) spaciousness affects emotions, which in turn affect moral judgments; (2) spaciousness is linked to our tolerant metaphor cognition, which leads to the change of moral judgment (Figure 1). Therefore, in the present study we explored the mediating effect of emotion between environmental spaciousness (spaciousness vs. narrowness) and moral judgment. We also evaluated the association between spaciousness and tolerant cognition by explicit and implicit measurements in present research. Experiment 3a used different graphics as priming stimuli to determine whether spaciousness triggers tolerant cognition for morality by explicit measurements. We proposed that spaciousness leads to tolerant moral judgment for moral behaviors. Experiment 3b tested the association using the IAT (an implicit measurement) and determined whether there were associations between spaciousness and tolerant cognition, and between narrowness and harsh cognition.

To better understand the impact of the environment on people’s moral judgment, we mainly examined the effect of spacious and narrow environments on moral judgment in the present study. Experiment 1 and Experiment 2 used an immersion task to explore the effect of environmental spaciousness on moral judgment and the mediator effect of emotion. We predicted that exposure to pictorial images of different degrees of spaciousness would lead to different moral judgments. Meanwhile, for the effect of environmental spaciousness, we tested the role of emotion (Experiment 1 and Experiment 2) and metaphor association (Experiment 3a and 3b) on the relationship between environment spacious degree and moral judgment.

## 2. Experiment 1

Experiment 1 was designed to test whether the spacious environment would lead to tolerant moral judgment compared to a narrow environment.

### 2.1. Method

#### 2.1.1. Design

Experiment 1a was a between-subject experimental design. The independent variable was the environmental spaciousness (spaciousness vs. narrowness). The dependent variable was rating scores of moral judgment.

#### 2.1.2. Participants

We determined the sample size for Experiment 1 by G*Power 3.1. The effect size of Experiment 1 was estimated to be small (η^2^ = 0.05). Using an α of 0.05 (two tailed) and a power of 0.95, the sample of Experiment 1 need 210. At last, a sample of 228 participants (50 males) aged between 18 and 25 years old with a mean age of 21.98 years (*SD* = 3.73) was recruited. Participants were sent a link to the online survey platform Sojump Survey (the spacious environment or the narrow environment) and in total we collected 113 records of a spacious environment and 115 records of a narrow environment, which met the minimum sample size. The Ethics Committee of South China Normal University permitted the protocol (SCNU-PSY-2020-4-050).

#### 2.1.3. Materials

**Figure 2:**
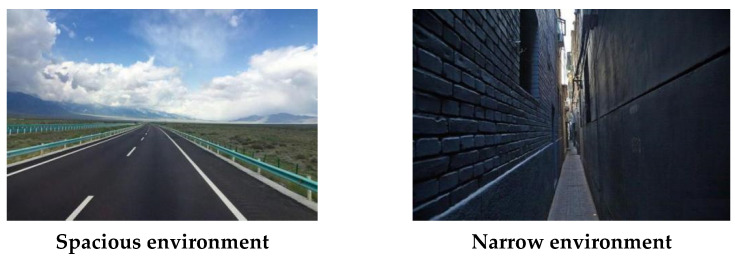
The photographs of spacious environment and narrow environment.

In order to ensure the authenticity of the environment, we chose to use the true photographs of the environment as environmental material. We conducted a search for spacious and narrow environments in Baidu’s gallery (a public and free website), most of which appeared as road environments, and other environments generally had inconsistencies in the degree of item richness. Therefore, in order to control the complexity and richness of the environment, we finally chose the spacious street environment and the narrow road environment in Experiment 1. Two environmental photographs with spaciousness and narrowness were selected from the free public archive at http://baidu.com/ (accessed on 20 November 2020). The images were cropped to fit a 500 ∗ 375 pixels frame using the Photoshop CS6 (Adobe Systems Software Ireland Ltd., Dublin, Ireland). For the materials of environments, a *t*-Test was used as statistical method to compare the result. The results supported the classification of the experimental material. Figure 2 shows the materials used in Experiment 1.

Considering the valid time of the immersion task, we only chose five stories often used in moral judgment. Five vignettes that characterized immoral behaviors were adopted from previous research, including eating pet dogs, taking bribes, and plagiarizing in a test, resume falsification, and wallet issues.

#### 2.1.4. Procedure

The participants were assigned to conduct an immersion manipulation task that involved viewing one of two pictorial images.

The participants read the following instructions:

“Imagine yourself in this environment. Looking around and seeing all aspects of your environment. Paying attention to the things of the environment and noticing the distance among things. Let yourself take in all the aspects of the environment in front of you”.

Participants were asked to rate the extent to which they felt as if they were in the place described on a 7-point scale (1 = not at all to 7 = very much). After completing the environmental immersion task, we required participants to use words to describe the feeling of immersion in the environment. Participants rated the spacious degree of the environments on a 7-point scale (1 = very narrow, 7 = very spacious). Finally, participants were asked to make moral judgment on a 9-point scale (1 = very immoral to 9 = very moral) for five vignettes.

Finally, participants were asked to report their awareness of experimental hypotheses. None of the participants were able to identify the purpose of the study accurately.

### 2.2. Result and Discussion

#### Manipulation Check

Independent Samples *t*-Test results showed that the main effect of environmental spaciousness was significant on environmental spacious degree and proved that operation of environmental spaciousness was valid, *p* < 0.001.

Table 1 shows the mean rating scores of moral judgment, emotion, immersion, and spacious degree in different environmental spaciousness. As expected, an Independent-Samples *t*-Test revealed no differences in the degree of immersion between spacious and narrow environments, *p* = 0.44. The results suggested that the rating scores of moral judgment (*p* = 0.03) were significantly higher in the spacious environment than in the narrow environment. The spacious environment leads participants to make more tolerant moral judgments compared to narrow environments. 

In Experiment 1 participants were instructed to imagine being in a narrow or spacious outdoor environment, and the result indicated that a narrow environment can predict more strict moral judgment. At the same time, some participants reported that the narrow environment of Experiment 1 have a lower brightness. The dim or darkness could lead participants to report a harsh moral judgment [13]. Therefore, the effect of environmental spaciousness in Experiment 1 might suffer from the interference of the brightness of environment. In order to further and better test this effect, we conducted Experiment 2. We also chose an indoor environment, which was manually manipulated (by its corresponding change in spaciousness) to undergo narrow treatment. This ensured that the interference of other factors could be excluded to the maximum extent possible in Experiment 2. Previous research found the role of emotion in the relation between environment and moral judgment [23,24]. So, we also explored the role of emotion in the relation between environmental spaciousness and moral judgment, anticipating that environmental spaciousness influences emotion, which leads participants’ moral judgment to be different in Experiment 2.

## 3. Experiment 2

### 3.1. Method

#### 3.1.1. Design

The design of Experiment 2 was the same as Experiment 1.

#### 3.1.2. Participants

We recruited a total of 122 (72 females) participants online. Participant ages ranged from 18 to 38 years (*M* = 22.37, *SD* = 4.27) and they were paid for their participation. The participants signed informed consent before the study. Sixty-seven people participated in the spacious environment condition and 55 in the narrow environment condition, meeting the minimum required sample size. We conducted G*Power 3.1 to estimate the effect size and power. The power was 0.78 and effect size d = 0.67. In addition, we obtained ethical approval from the Ethics Committee of South China Normal University under protocol SCNU-PSY-2020-4-050.

#### 3.1.3. Materials

**Figure 3:**
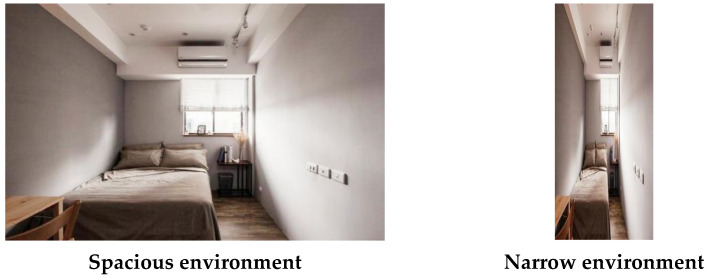
The phonographs of spacious environment and narrow environment.

Besides the materials of Experiment 1, we also chose other environmental images. Environmental photographs with spaciousness were selected from the free public website, http://baidu.com/ (accessed on 16 December 2020). An image of a spacious environment was cropped to fit a 770 × 481-pixel frame. The same image was used for the narrow environment but cropped to fit a 200 × 481-pixel frame using Photoshop CS6 (Adobe Systems Software Ireland Ltd., Dublin, Ireland; see Figure 3). For the materials of environments, a Student’s *t*-Test was used to compare the results. 

#### 3.1.4. Procedure

The procedure was the same as the Experiment 1. In Experiment 2, however, after the immersion task we required participants to use words to describe the feeling of immersing themselves in the environment. We also measured the emotion of participants when facing the environment on a 7-point scale (1 = very negative, 7 = very positive). 

### 3.2. Result and Discussion

#### 3.2.1. Manipulation Check

Independent Samples *t*-Test results showed that the main effect of environmental spaciousness was significant on the environmental spacious degree and proved that the operation of environmental spaciousness was valid, *p* < 0.001.

Table 2 shows the mean rating scores of moral judgments in different environmental spaciousness. As expected, the Independent Samples *t*-Test revealed no differences in the degree of immersion between spacious and narrow environments, where *p* = 0.13. The results suggested that the rating scores of moral judgments (*p* = 0.004) and emotion (*p* < 0.001) were higher in the spacious environment than in the narrow environment. The spacious indoor environment leads to more tolerant moral judgment compared to narrow indoor environments. This effect of environmental spaciousness on moral judgment still exists in the indoor environment.

#### 3.2.2. The Intermediate Effect of Emotion

There was a positive relationship between emotion and moral judgment. We further tested whether emotion would account for the influence of environmental spaciousness on moral judgment. To explore the mediating effect, the data were analyzed according to Stepwise analysis using the Statistic Package for Social Science [42]. We also used Pearson’s correlation analysis to explore the relationship among environmental spacious degree, emotion, and moral judgment. Pearson’s correlation analysis results indicated that emotion was positively associated with the environmental spaciousness degree and moral judgment (*p* < 0.01).

**Figure 4:**
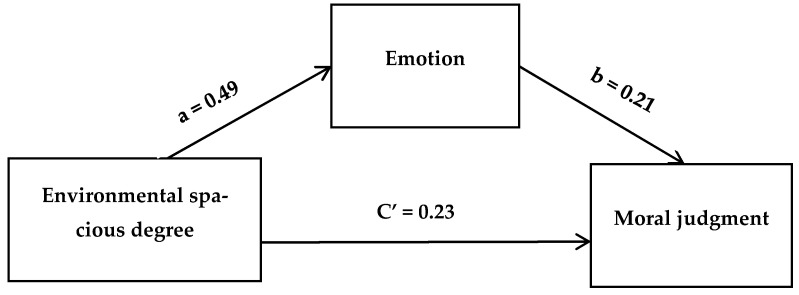
The role of emotion in the relation between environmental spacious degree and moral judgment (a represents the regression coefficient when spaciousness predicts emotional scores; b represents the regression coefficient when mood score predicts moral judgment score; C’ represents the effect of spaciousness on moral judgments after considering emotions).

Results of the mediating effect analysis showed that emotion mediated the relation between environmental spacious degree and moral judgment (Table 3). We also used stepwise analysis to explore the intermediary effect of emotion. The value of ab/c is the mediating effect as a percentage of the total effect. The indirect effect of environmental spaciousness degree on moral judgment via emotion was significant, where ab/c = 0.45 (Figure 4). In other words, the result showed that emotion was a significant mediator (*p* < 0.05). The mediation results suggest that emotions partially mediate the effect of the environmental spacious degree on moral judgment.

Consistent with previous research, the results proved the role of emotion between environments and moral judgment [6,22,41,43]. However, the effect of environmental spaciousness on moral judgment is only partially influenced through emotion. 

Previous research also found that metaphor has an important role in the relationship between the environment and morality. Before exploring the effect of metaphor, we firstly tested the activating meaning of different environments using word clouds.

## 4. The Word Clouds

**Figure 5:**
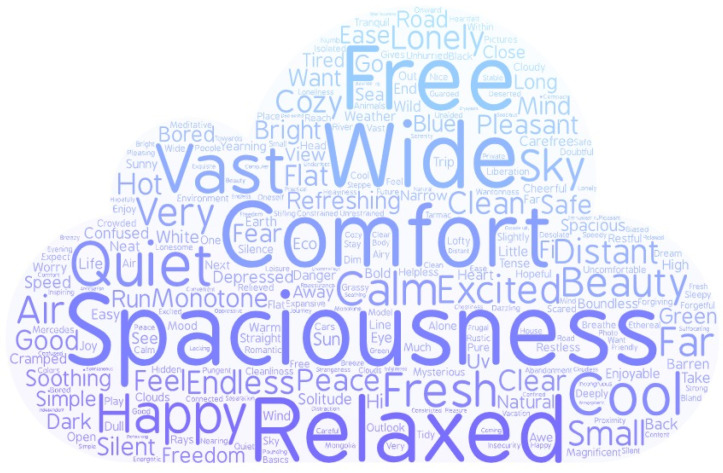
The word clouds in the spacious environment.

**Figure 6:**
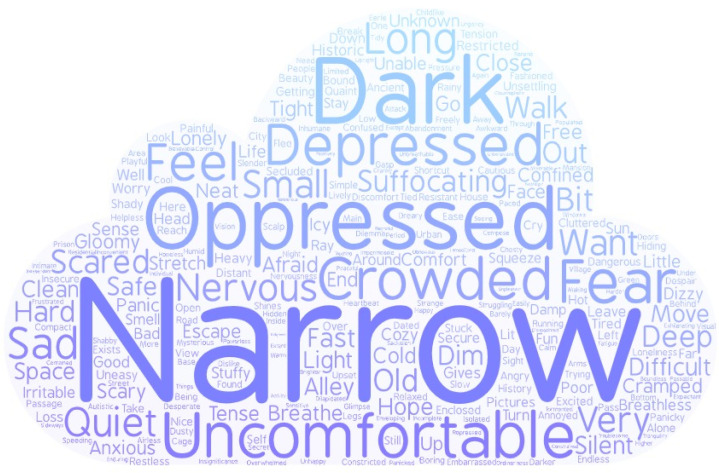
The word clouds in the narrow environment.

In Experiment 1 and Experiment 2, participants all were asked to describe the feeling of immersing themselves in a certain environment using the words. We used the analysis of the word clouds to test the words in different environments. Firstly, we obtained the frequency of the different words describing the feeling in the spacious environment and in the narrow environment according to the website (http://www.picdata.cn/picdata/ci_b.php, accessed on 10 September 2021). Further, these data were input to the online website of the word cloud (https://wordart.com/, accessed on 10 September 2021). The website could generate word clouds. The size of words in a word cloud indicated the frequency of the words. When the words occurred more frequently, the size of the words was bigger. The word clouds of different feelings in the spacious environment and the narrow environment can be seen in Figure 5 and Figure 6.

The result of the word clouds showed that the priming effect of environmental images is reasonable. When individuals faced a spacious environment in Experiment 1 and Experiment 2, they activated the feeling of spaciousness, and participants facing narrow environments activated the feeling of narrowness. We also found that participants have more positive awareness of exposure to the spacious environment compared to exposure to a narrow environment. These results were consistent with previous research supporting that different environments could express different thoughts [44,45]. The spacious environment always referred to the wide and big environment, and the narrow environment was defined as the environment of narrowness and smallness. The highness or bigness of space activated more positive conception (bigness is good or bigness is power) and emotion [44].

In general, the environmental spaciousness leads to us triggering different emotions and different meanings. 

Previous research has also found that metaphor plays an important role in the relationship between the environment and morality. According to our results, we also found that spacious and narrow environments have activated different meanings (spaciousness vs. narrowness). According to the conception of spaciousness and space metaphor, we proposed that the conception of spaciousness or width is related to tolerant cognition. The effect of environmental spaciousness on moral judgments may be due to the activation of spaciousness or narrowness triggering tolerant or harsh cognitive attitudes. We also found the environmental spaciousness activated the conception of width according to the word cloud. Experiment 3 mainly tested the metaphor association between the conceptions of width and tolerant cognition using different graphics which activated the conception of width and narrowness.

## 5. Experiment 3

### 5.1. Experiment 3a

Experiment 3a adopted a 2 (graphics type: width vs. narrowness) × 2 (behavioral type: moral vs. immoral) within-subject experimental design. Participants were required to make moral judgment for the same behaviors using the same photographs. The dependent variables were rating scores of moral judgment.

#### 5.1.1. Method

##### Participants

We determined the sample size for Experiment 3a by G*Power 3.1. The effect size of Experiment 1 was estimated to be small (η^2^ = 0.05). Using an α of 0.05 (two tailed) and a power of 0.95, study participants for Experiment 3a were 36. At last, 40 participants, ranging from 18 to 24 years (24 females; *M* age = 20.34, *SD* = 2.58) were recruited. The study was approved by the Institute Ethics Committee of South China Normal University (SCNU-PSY-2020-2-006).

##### Materials

**Figure 7:**
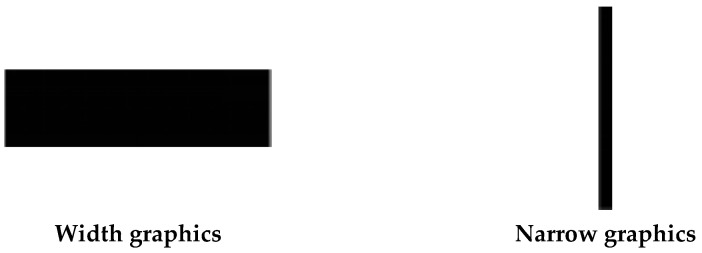
Examples of graphics as priming images in Experiment 3a.

Width and narrowness in Experiment 1 were activated by wide graphics and narrow graphics. Stimuli included two types of pictures, namely wide graphics and narrow graphics. The fill color was black. Wide graphics were 1024 × 300-pixel while narrow graphics were 28 × 500-pixel (Figure 7).

**Figure 8:**
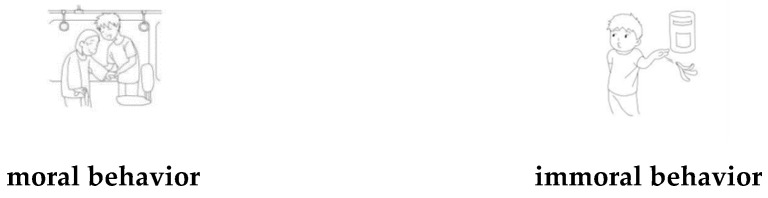
Example of materials used in the Experiment 3a.

Scene drawings for moral and immoral behavior were the same as in previous research [43]. Samples of the materials for this experiment are shown in Figure 8.

##### Procedure

**Figure 9:**
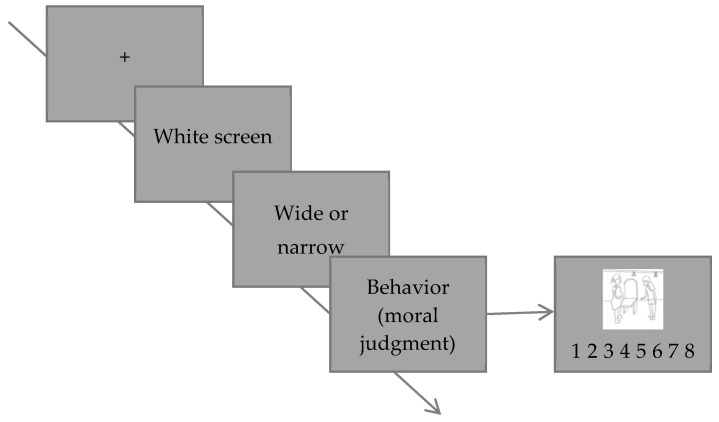
Example of event sequences on moral judgment task in Experiment 3a.

Stimuli were presented against a white background in the center of a 1700CRT monitor (1024 × 768 resolution, 100-Hz refresh rate). Each trial was initiated by a presentation of fixation cross ‘+’ for 500 ms followed by a 300 ms blank screen. Then, one image for 3000 ms appeared, followed by a blank screen 100 ms. Then, target images were presented until pressing the keyboard. Participants were instructed to make judgments for scene drawings of moral beauty on a 7-point scale (1 = Extremely moral ugliness, 7 = Extremely moral beauty). Experimental procedures are shown in Figure 9.

#### 5.1.2. Result and Discussion

Each participant’s mean rating scores of moral judgment for scene drawings of moral and immoral behaviors were calculated. Data beyond three standard deviations from the mean value were excluded from further analyses. The data were not normally distributed (*p* < 0.05). Therefore, data were normalized for comparisons of differences. Data were entered into a 2 (graphics type: width vs. narrowness) by 2 (behavioral type: moral vs. immoral) repeated measures analysis of variance. 

**Figure 10:**
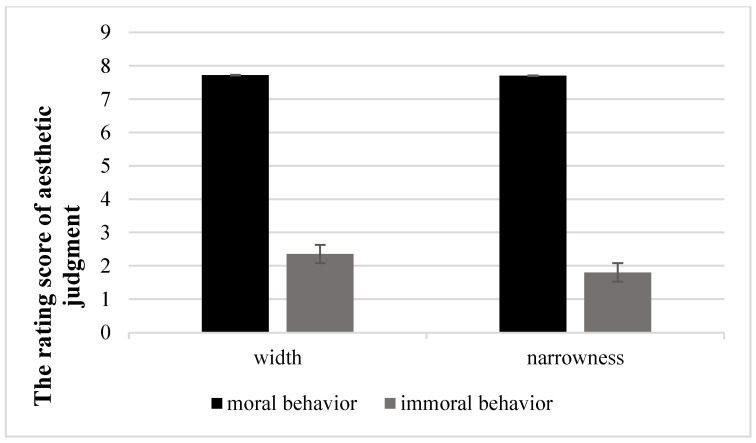
The interaction effect between graphics and behavior.

Table 4 shows mean ratings of moral judgment under different conditions in Experiment 1a. Moral rating scores revealed that the main effect of graphics type was significant, *F* (1, 39) = 40.66, *p* < 0.001, η^2^ = 0.51. In the width condition (5.03 ± 0.068), scene drawings of morality were perceived as higher than in the narrowness condition (4.75 ± 0.09). Main effect of behavioral type was significant, *F* (1, 39) = 513.50, *p* < 0.001, η^2^ = 0.93, and interactions between graphics type and behavioral type were also significant, *F* (1, 39) = 46.23, *p* < 0.001, η^2^ = 0.54 (Figure 10). Aa simple effects test revealed that for scene drawings of immoral behaviors, individuals in width and narrowness conditions exhibited a significant difference in moral judgments (*p* < 0.05), but the difference was not significant for scene drawings of moral behaviors (*p* > 0.05).

Experiment 3a revealed that the moral judgment of scene drawings regarding moral was higher than the judgment regarding immoral, which corresponded to the result of the material evaluation, and proved the credibility of moral materials. Moreover, the results also provide evidence for the association between width and cognitive judgment, that the conception of width activated tolerant moral judgment. Experiment 3b further explored the associations.

### 5.2. Experiment 3b

This experiment aimed at evaluating the association between spaciousness and tolerant cognition using IAT.

#### 5.2.1. Method

##### Participants

A total of 30 participants aged between 18 and 24 years (19 females) were recruited. Ethical approval for this study was obtained from the Institutional Ethics Committee of South China Normal University (SCNU-PSY-2020-2-006).

##### Materials

The width and narrowness stimulus were the same as those used in Experiment 1. Hence, 17 words were used to describe tolerant and harsh cognition. Words depicting tolerant and harsh cognition were selected by searching for the synonyms and antonyms of ‘宽松’ (kuan1song1) and ‘严厉’ (yan2li4). A separate group of 11 participants rated the degree of tolerant or harsh cognition, familiarity and comprehensibility on a 7-point scale. ‘1′ indicated ‘extremely harsh’ and ‘7′ indicated ‘extremely tolerant’. The results of the two sets of materials showed significant differences in tolerance (*p* < 0.05), but no significant difference in terms of familiarity and comprehensibility (*p* > 0.05) (See Table 5). The samples of the materials used in this study are shown in Figure 2.

In Experiment 2, wide graphics matched with words depicting tolerant cognition while narrow graphics matched with words depicting harsh cognition. They constituted a compatible combination. Wide graphics and words depicting harsh cognition and narrow graphics and words depicting tolerant cognition formed incompatible joint discrimination tasks.

##### Procedure

Each participant completed a total of 5 classification tasks: 1—single categorization for target (wide graphics/narrow graphics; 20 trials); 2—single categorization for implicit association (words describing tolerant cognition/words describing harsh cognition; 17 trials); 3—combined categorization task, with practice and data collection trials (wide graphics + words describing tolerant cognition/narrow graphics + words describing harsh cognition; 37 trials); 4—single categorization of target concept (as block 2), but with reversal of the side of the screen on which the category was presented to that which the picture needed to be categorized (20 trials); 5—combined categorization task, with practice and data collection trials (as block 3) but reversed categorization of target categories (narrow graphics + words describing tolerant cognition/wide graphics + words describing harsh cognition; 37 trials). Only data from tasks 3 and 5 were used for analysis. 

Participants completed an implicit association test (IAT) measuring implicit association between graphics and words describing different cognition. The IAT task was completed using IBM-compatible desktop computers, using the E-prime program. At the center of the computer screen, the stimuli to which participants had to ascribe to one of two (or four) categories were randomly presented. Participants responded to categorization task by pressing either the ‘E’ key with the left index finger or the ‘I’ key on the numeric keypad with the right index finger. The meanings of the keys were shown in Table 6 in different tasks.

#### 5.2.2. Result and Discussion

We used G* Power 3.1 to estimate the power (1 − β = 0.75) and effect size d = 0.68 getting to the medium level. In the data reduction procedure, 300 was recorded if less than 300 ms, 3000 if greater than 3000 ms, and deleted if the error rate exceeded 20% [44]. In this study, no data were excluded from the analysis because of an error rate lower than 20%. Data were not-normally distributed (*p* < 0.05). Therefore, they were normalized for comparisons of differences.

We compared categorization of wide graphics and narrow graphics paired with words depicting tolerant and harsh cognition. Table 7 shows mean accuracy (ACC) and reaction time under different conditions. The finding provides a measure of implicit attitudes towards the two categories. Quicker reactions for one category indicated a more positive implicit attitude towards that category.

Paired sample *t*-Test analysis revealed that participants had significantly shorter reaction times when wide graphics were paired with words describing tolerant cognition, than when narrow graphics were paired with words describing harsh cognition, *t* (29) = −6.489, *p* < 0.001, Cohen’s *d* = 1.19, 95%CI [−0.098, −0.051]. There was no significant difference between the condition in which wide graphics were paired with words describing tolerant cognition and the condition in which wide graphics were paired with words describing harsh cognition, *t* (29) = −0.36, *p* = 0.721, 95%CI [−0.0091, 0.0064].

##### IAT Effect

From the result of different parts of joint discrimination task, calculated D was 1.19. D-scores generally fell between −2 and 2, and they were tested against 0 to determine whether there was evidence of an association (with a value of 0 reflecting no difference in strength between the pairs of association measured by the two tasks) [46,47]. In Experiment 2, D value was found to be 1.19, implying a significant IAT effect. Therefore, there was a strong implicit association between wide graphics and words describing tolerant cognition as well as between narrow graphics and words describing harsh cognition.

Moreover, it was found that there was an implicit association between width and tolerant cognition. When wide graphics were paired with words describing tolerant cognition, the reaction time of participants was shorter than that when wide graphics were paired with words describing harsh cognition. These findings were consistent with findings of Experiment 3a, providing further evidence of the relation between width and tolerant cognition, and between narrowness and harsh cognition.

## 6. General Discussion

### 6.1. The Effect of Environmental Spaciousness on Moral Judgment

The present research explored the effect of different environments on moral judgment. Through two experiments, we found that, compared with the narrow outdoor environment, spacious outdoor environment exposure can predict more tolerant moral judgment (Experiment 1), and this effect still exists after exposure to the indoor environment (Experiment 2). These results also supported the effect of physical environment on moral judgment [6,8] and provide a new angle for the physical environment on moral judgment.

### 6.2. The Intermediary Role of Emotion

The results proved the mediating effect of emotion between environments and moral judgment. The results of the word cloud also revealed that the positive conceptions (e.g., freedom, happiness, comfort) can be activated when in a spacious environment, and the negative conceptions (e.g., repression; strictness; fear; oppression) were activated when in a narrow environment. So, the spacious environment triggered a higher positive emotional state compared with the narrow environment, which led to tolerant moral judgment compared to a narrow environment. These results were consistent with previous findings that proved that emotion affected moral judgment [48,49,50,51,52]. Positive emotion urged participants to make tolerant moral judgment [23,53]. Negative emotion was negatively associated with moral judgment, and higher negative emotion with more harsh moral judgment [6].

The emotion led participants to have different active thinking and different ability to make decisions [54,55,56,57]. Individuals in a positive emotional state used simple conception or a lower level of cognition to reason and make a moral judgment, compared with individuals immersed in a sad emotional state. So, a spacious environment activated the positive emotion which led participants to make a decision using simple cognition (lower level of cognition) compared to a narrow environment. In general, emotion influenced the relationship between environmental spaciousness and moral judgment.

### 6.3. The Role of Metaphor Association

Experiment 3a revealed that participants scored higher on moral judgment in the wide priming condition compared to the narrow condition. Experiment 3b further tested the association between the conception of spaciousness and tolerant cognition by implicit measurement and revealed that there was metaphor association between the concept of spaciousness and tolerant cognition, as well as between the concept of narrowness and the harsh cognition. These findings are consistent with our hypotheses and provide some evidence for the effects of metaphors in cognitive judgment [33,58]. Because the origin domain of metaphor comes from the body’s perceptual-motor system and the experience of environmental interaction, the rich experiences (somatic sensations, proprioception, spatial relational shapes, and kinesthetic manipulation experiences) gained by the body in its continuous interaction with the spatiotemporal environment become the origin structure for the formation of metaphor. The spaciousness of the environment implies big areas and large space bearing capacity, which is associated with tolerant cognition. This is consistent with metaphor use in different cultures. For example, the English phrase ‘wide-minded’ and Chinese words, ‘kuan1yi3dai4ren2’ describe tolerant cognition. in Chinese. The association between them is stored in our long-term memory and metaphor recall influences cognition [25,59]. Finally, when the concepts of spaciousness or narrowness were activated by different environment stimuli, this triggered the metaphor association of tolerance, which in turn led to more tolerant moral judgments than participants in the narrow environment.

### 6.4. Limitations and Future Direction

In this study, there are some shortcomings, such as the selection of environmental materials. For the environmental materials in Experiment 1, some participants reported that the narrow environment had a lower brightness, and participants may have considered those pictures as belonging to urban and non-urban environments, which might confound the environmental spaciousness effect on moral judgment. In Experiment 2, we balanced the brightness and the effects caused by the different objects within the environment by using non-real environmental picture. Even though we verified its realism before the experiment, there may still be participants who may have a sense of non-realism for the environmental picture, which may affect the individual’s cognition and judgment. In future studies, we would look for more reasonably spacious and narrow environments to exclude these confounding effects, such as using the true environment or VR to explore the effect of environmental spaciousness on moral judgment, which could also improve experimental validity. 

In addition, the current study enrolled only Chinese people as participants. In the future, we could sample Western participants to test the role of metaphor in the relation between environmental spaciousness on moral judgment. In addition, in this study, we mainly focused on students, and did not investigate the effects of education level, occupation, place of residence, and nationality, which may affect the diversity of participants to some extent and weaken the external validity. In future studies, we can investigate whether the effect of environmental spaciousness on moral judgment changes across different types of participants to increase the diversity and external validity of the study.

The researchers showed that moral behaviors are related to moral judgment [4,60,61,62]. According to the results of the present research, moral judgment is different between spacious and narrow environments. Future research can explore the effect of a spacious environment on moral behaviors.

## 7. Conclusions

The present research explored the influence of environmental spaciousness on moral judgment. Meanwhile, we explained the effect of environmental spaciousness from two angles, namely emotion and metaphor. The spacious environment activated positive emotion and the metaphor of tolerant cognition, which further influenced moral judgments. These results extend evidence of the relation between the physical environment and moral judgment. The findings also call for further attention to be directed to the effect of environmental design and provide suggestions for reducing the negative effect of narrow environments.

## Figures and Tables

**Table 1:** The Mean rating score of moral judgment in different conditions.

Environmental Spaciousness	Spaciousness	Narrowness	*t*	*p*	95%CI		Cohen’s *d*
					low	high	
Moral judgment	2.51 ± 1.18	2.22 ± 0.77	2.20	0.03	0.03	0.55	0.29
Immersion	5.16 ± 1.01	5.05 ± 1.07	0.78	0.44	−0.17	0.38	
Spacious degree	5.44 ± 1.27	2.40 ± 1.30	17.87	<0.001	2.71	3.38	2.37

**Table 2:** The Mean rating score in different conditions.

Environmental Spaciousness	Spaciousness	Narrowness	*t*	*p*	95%CI		Cohen’s *d*
					Low	High	
Emotion	4.97 ± 1.48	3.93 ± 1.33	4.06	0.001	0.53	1.55	0.74
Moral judgment	2.78 ± 1.25	2.16 ± 1.01	2.98	0.004	0.21	1.04	0.55
Immersion	4.73 ± 1.41	5.13 ± 1.17	−1.68	0.096	−0.88	0.072	
Spacious degree	4.73 ± 1.82	2.44 ± 1.61	7.30	0.001	1.67	2.92	1.33

**Table 3:** The correlation between emotion, environmental spacious degree and the moral judgment.

	Environmental Spacious Degree	Moral Judgment
Emotion	0.49 **	0.28 **
Moral judgment	0.21 *	

Note: ** stands that correlation is significant at the 0.01 level (2-Tailed); * stands that correlation is significant at the 0.05 level.

**Table 4:** Mean rating of moral judgment under different conditions (M ± SD).

Behavior	Graphics	Moral Judgment	
		*M*	*SD*
Moral	Width	7.72	0.13
Moral	Narrowness	7.70	0.15
Immoral	Width	2.35	0.76
Immoral	Narrowness	1.80	0.66

**Table 5:** The mean rating scores of the words describing different cognition.

	Tolerant Cognition	Harsh Cognition	*t*	*p*	95%CI		Cohen’s *d*
					low	high	
Tolerance	4.00 ± 0.60	1.99 ± 1.20	5.43	<0.001	1.19	2.84	1.63
Familiarity	4.09 ± 0.82	3.74 ± 0.96	2.07	0.07	−0.03	0.73	
Comprehensibility	3.60 ± 0.61	3.65 ± 1.03	−0.20	0.84	−0.55	0.46	

**Table 6:** The meaning of the keys in different tasks.

Task	“E” Key	“I” Key
1	width	narrowness
2	tolerant cognition	harsh cognition
3	width/tolerant	narrowness/harsh
4	harsh cognition	tolerant cognition
5	width/harsh	narrowness/tolerant

**Table 7:** The mean ACC and reaction time under different conditions.

	ACC		Reaction Time	
	*M*	*SD*	*M*	*SD*
Compatibility	0.950	0.047	581.02	91.45
No-compatibility	0.952	0.042	695.36	176.64

## Data Availability

The data presented in this study are available on request from the corresponding author. The data are not publicly available due to restrictions e.g., privacy or ethical.
